# An ingestible origami-inspired metamaterial for prolonged oral delivery of therapeutics

**DOI:** 10.1038/s41467-026-76028-y

**Published:** 2026-07-27

**Authors:** Farhad Javid, Sahab Babaee, Jasmine Quigley, Ameya Kirtane, Hormoz Mazdiyasni, Jaimie Rogner, Cody Cleveland, Taylor Bensel, Vance Soares, Joy E. Collins, Kaitlyn Hess, Shane McDonnell, Alison M. Hayward, Robert Langer, Giovanni Traverso

**Affiliations:** 1 Department of Chemical Engineering and Koch Institute for Integrative Cancer Research, Massachusetts Institute of Technology, Cambridge, MA USA; 2 Department of Mechanical Engineering, Massachusetts Institute of Technology, Cambridge, MA USA; 3 Division of Gastroenterology, Brigham and Women’s Hospital, Harvard Medical School, Boston, MA USA; 4 Institute for Medical Engineering and Science, Massachusetts Institute of Technology, Cambridge, MA USA; 5 Division of Comparative Medicine, Massachusetts Institute of Technology, Cambridge, MA USA

**Keywords:** Drug delivery, Translational research, Materials for devices

## Abstract

Poor adherence to oral drug regimens adversely affects treatment outcomes and contributes substantially to preventable healthcare costs, underscoring the need for long-acting drug delivery systems that sustain therapeutic levels over extended periods. To address this, we developed an origami-inspired, oral drug delivery dosage form capable of prolonged residence in the gastric cavity. This system achieves ease of administration and extended gastric residency through geometric design and shape optimization, allowing the dosage form to fold inside a standard ingestible capsule. Upon the capsule’s dissolution in the stomach, the dosage form rapidly transforms into its deployed configuration. Our formulation and manufacturing techniques enable the development of a long-acting dosage form with up to 40% weight loading capacity of sample drugs, such as moxifloxacin. The high surface-area-to-volume ratio of the origami design enables approximately linear release kinetics over the observed time window of candidate drugs. We incorporated pH-sensitive elements that dissolve in the neutral portions of the GI tract into the dosage form to promote its dissociation and safe transit in the intestine. In vitro and in vivo studies using swine models demonstrated extended gastric residency of up to three weeks (with a minimum of one week) and an approximately constant release rate for three days. Endoscopic and radiographic evaluations of the animal models confirmed that the dosage forms safely pass through the intestine without causing gastrointestinal obstruction or injury.

## Abstract (web summary)

Poor adherence to oral drug regimen adversely affects treatments. Here, the authors developed an origami-inspired, oral drug delivery dosage form capable of prolonged residence in the gastric cavity.

## Introduction

Poor patient adherence to therapies reduces treatment efficacy and contributes to over 100 billion dollars per year in preventable hospitalizations in the US alone^1^. Extended drug release technologies, which enable drugs to be delivered over the course of days to months through a single administered dose, can significantly improve patient compliance and drug effectiveness^2,3^. For oral therapies, one of the main challenges in achieving extended delivery ( > 1–2 days) is the limited residence time of drugs in the gastrointestinal (GI) tract^4^. As such, several oral drug delivery dosage forms have been developed to achieve prolonged GI residency using various strategies including floating^5^, swelling^6,7^, adhesion to the mucosal layer^8^, and shape transformation^9,10^. While most of these techniques achieve around a day-long residence in the GI tract with limited drug loading capacity, shape-transformable systems are shown to achieve multi-day residence with drug loading capacities ranging between 20 and 40 w/w % of the system^9,11,12^. Multiple design objectives, including easy delivery and deployment, prolonged retention, sustained release, and safe removal of dosage forms, are often achieved by effectively optimizing a multi- component shape-transformable system. The multi-component systems, however, are complex, have a high cost of manufacturing, may need special delivery procedures, and are often tuned to deliver specific drug types. Previous attempts to design generic dosage forms made of a single material and capable of carrying different drug types have resulted in limited GI residence (∼1 day) in animal models and humans^13^. Here, we propose a dosage form, designed based on a donut-shaped origami geometry, that provides the device with both mechanical strength and the ability to carry drugs. Additionally, the thin paper-like structure of the origami design provides a high surface-area-to-volume ratio that supports approximately linear release kinetics over the observed time window and minimizes the risk of GI tract blockage.

Origami, or the Japanese art of paper folding, is a flexible technique for developing shape-transformable structures^14–17^ for a wide array of complex engineering applications including robotics^18^, optics^19^, communications^20^, aerospace^21^, acoustics^22^, soft electronics^23^ and medicine^24,25^. Through careful design of periodic fold patterns, i.e. creases, origami structures can attain properties like extreme compactness and controlled expansion^26–31^. Additionally, when coupled with advanced manufacturing processes, origami design can convert thin sheets into mechanically robust shape-shifting and programmable metamaterials^32,33^. In these metamaterials, both shape and mechanical properties are governed by the folding angles along creases, tailored to achieve specific characteristics^34–38^.

Inspired by circular Miura-ori origami^29,39,40^ – a periodic tessellation of parallelograms – we present a highly reconfigurable shape-shifting metamaterial, whose shape can be drastically transformed from a compact semi-cylinder in the folded state to a three-dimensional (3D) torus by releasing the elastic energy stored within the creases. We demonstrate that such origami metamaterial made of drug-loaded polycaprolactone (PCL) sheets can be used as an oral drug dosage form, delivered using standard ingestible capsules. The origami-inspired dosage form (ODF) is orally delivered to the gastric cavity and self-deployed to a toroidal shape, enabling prolonged gastric residence while releasing drug depots. Through a combination of analytical and experimental methods, we designed a dosage form that maximizes the drug-loading capacity of the system, guarantees the safety of the oral delivery via standard ingestible capsules, and extends the device residence in the gastric cavity to several days. Our in vitro animal studies demonstrated that such a device can reside inside the stomach for a minimum of one week while releasing a sample drug, e.g. moxifloxacin, for 3 days into the animal body. Based on these findings, we identify how the ODF may serve as a class of extended gastric-residence systems for drug delivery. Such systems may improve treatment adherence (the likelihood that patients follow a prescribed medication regimen) by reducing dosing frequency, particularly for therapies that require sustained drug exposure over multiple days.

Unlike prior gastric-resident systems that rely on multi-component expandable architectures composed of rigid arms, elastic elements, or assembled mechanical components, the present design introduces a fundamentally different approach based on origami metamaterials. This enables single-material fabrication, geometrically programmed deployment, and a continuous design space for tuning mechanical and functional properties. By embedding functionality within the geometry of a continuous structure, this approach reduces structural complexity while preserving key performance requirements, including compact encapsulation, reliable deployment, and prolonged retention.

## Results

### Design of shape transformable origami-inspired metamaterials

**Fig. 1:**
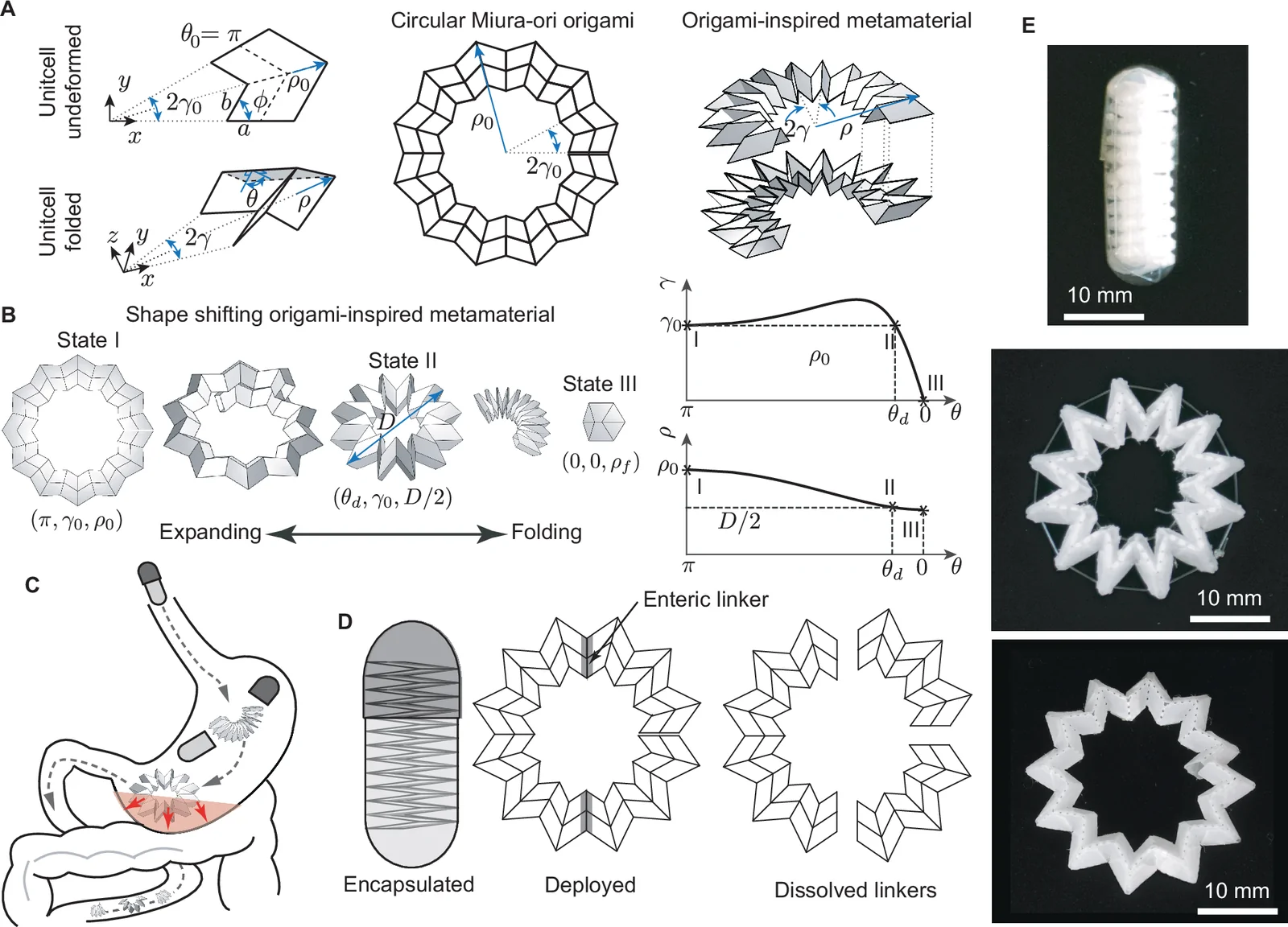
Shape-shifting origami-inspired metamaterial. **A** The undeformed (top left) and folded configurations (bottom left) of the unit cell that can be circularly arranged to form a circular Miura-ori origami (middle). The design parameters are the edge lengths, the angle between them, and the central angle, denoted by a, b, and φ, and, ${\gamma }_{0}$, respectively. The metamaterial (right) is formed by joining two Miura-ori circular patterns. **B** Sequence of progressively deformed configurations of the metamaterial characterized by $(\theta,\,\gamma,\,\rho )=(\pi,\,{\gamma }_{0},\,{\rho }_{0})$ for undeformed flat, *state I*, $({\theta }_{d},\,{\gamma }_{0},\,D/2)$ for deployed/expanded, *state II*, and $(0,\,0,{\rho }_{f})$ for fully folded, *state III*, configurations, where θ is the folding angle between radially adjacent facets and ρ the outer radius of the origami. ${\rho }_{f}$ is defined at the limit as $\rho {|}_{\theta \to {0}^{+}}$ (see Geometric characterization of circular Miura-ori origami in the Supporting Information) while ${\theta }_{d}$ and D are the folding angle and the outer diameter of the metamaterial at the deployed configuration. An intermediate configuration between *state I* and *state II*, in which the two ends of the metamaterial pass each other, is also shown to better illustrate the folding sequence. The plots show the variation of ρ and γ as a function of θ between the states. **C** Schematic of oral administration and deployment of the encapsulated metamaterial dosage form in the stomach. The red arrows show the drug dissolution into the gastric fluid. **D** Schematics of the dosage form in its encapsulated form, deployed in the stomach, and dissociated in the intestine environment are presented. The metamaterial dosage form is made of drug-loaded polymer films with two embedded pH-sensitive linkers. **E** A representative dosage form prototype shown in the encapsulated and deployed configurations.

Miura-ori^11^ fold pattern has been commonly used in design of engineering and architectural structures^37,38,41^ (see Miura-ori fold pattern in Fig. S1 in the Supporting Information). Variants of Miura-ori pattern are also suggested for generating structures with nonzero curvatures^42–44^ and complex transformable properties. Circular (tapered^29^) Miura-ori is one such structure, created by inclining the straight creases of a planar Miura-ori into the radii of a circle, which forms a polar, rather than parallel, pattern. A schematic of the circular Miura-ori unit cell, showing the undeformed/flat and deformed/folded configurations, is illustrated in Fig. 1A. The geometry of the undeformed unit cell is characterized by four geometric parameters: the unit cell edge lengths (a and b), the angle between these two edges (φ), and the central angle of the unit cell $({\gamma}_{0})$. The unit cells are circumferentially stacked to form a circular Miura-ori origami (see Fig. 1A-middle). The origami-inspired metamaterial, suggested here for the dosage form, is a 3D toroidal structure^40^ formed by fusing two circular Miura-ori origamis as shown in Fig. 1A-right.

The origami-inspired metamaterial is a highly foldable shape-shifting structure that transforms from an undeformed planar shape to a fully folded configuration. Different transformation stages are shown in Fig. 1B. Starting from the undeformed flat configuration (the most left inset on Fig. 1B), the two circular Miura-ori sections of the metamaterial fold in opposite directions and the metamaterial expands into a 3D toroidal shape. Initially, the structure expands in the circumferential direction; the open ends of the metamaterial then overlap, as can be seen in the second inset on Fig. 1B. In this stage, the origami-inspired metamaterial shows a semi-auxetic behavior in which it expands in circumferential and out-of-plane directions. However, if the folding continues, the structure shrinks circumferentially into a full (the third inset on Fig. 1B) and then a partial (the fourth inset on Fig. 1B) 3D toroidal shape. Further folding leads to a fully folded stage (the fifth inset on Fig. 1B) where the facet of a single unit cell collapses on each other and all unit cells of the metamaterial are stacked in circumferential direction. We named the undeformed stage of the structure as *state I*, the full toroidal shape stage as *state II*, and the fully-folded stage as *state III*. We also note that the central angle, γ, of each unit cell at *state II* equals that of the undeformed structure, ${\gamma }_{0}$. The outer diameter, D, and the folding angle, ${\theta }_{d}$, at *state II* are determined by solving the geometric equations of a unit cell where ${\gamma=\gamma }_{0}$ (see Geometric characterization of circular Miura-ori origami in the Supporting Information).

In contrast, when the metamaterial expands from the fully folded state (*state III*), the transformation into *state I* becomes infeasible. This is because the two open ends of the structure meet at *state II*, preventing the metamaterial from collapsing into the undeformed *state I*. In our design, *state II* is the final and stable state of the origami-inspired structure when it is released from the fully-folded *state III*. The variations of the metamaterial’s center angle, γ, and its outer radius, ρ, as a function of the folding angle, θ, are plotted in Fig. 1B-right.

Our origami dosage form (ODF) is orally administered by being folded into a standard 000-size ingestible capsule, shown in Fig. 1C. Upon reaching the stomach, the gelatin capsule dissolves and the ODF quickly expands to its deployed shape with a diameter larger than the pylorus. The 3D toroidal geometry of the ODF prevents passage through the pylorus and provides prolonged residence in the stomach for several days while releasing the drug. Drug release occurs through erosion and partial degradation of the thin, drug-loaded polymeric sheets that comprise the ODF, allowing the drug to diffuse out gradually. This degradation results in a reduction of the ODF’s mechanical strength and its eventual passage into the intestine. To ensure the ODF’s safe passage through the GI tract, two bands of pH-sensitive elements, which dissolve in the near neutral pH environments of the intestine, are incorporated into the ODF. Figure 1D display the schematics of the ODF geometry when it is encapsulated inside an ingestible capsule, deployed in the stomach, and dissembled in the intestine. Representative drug-loaded ODF prototype inside a 000-gelatin capsule (top) and deployed (middle and bottom) are shown in Fig. 1E. Two surgical sutures are passed through the ODF to ensure that its open ends remain locked after deployment and through gastric residence.

### Geometric characterization of the origami dosage form (ODF)

**Fig. 2:**
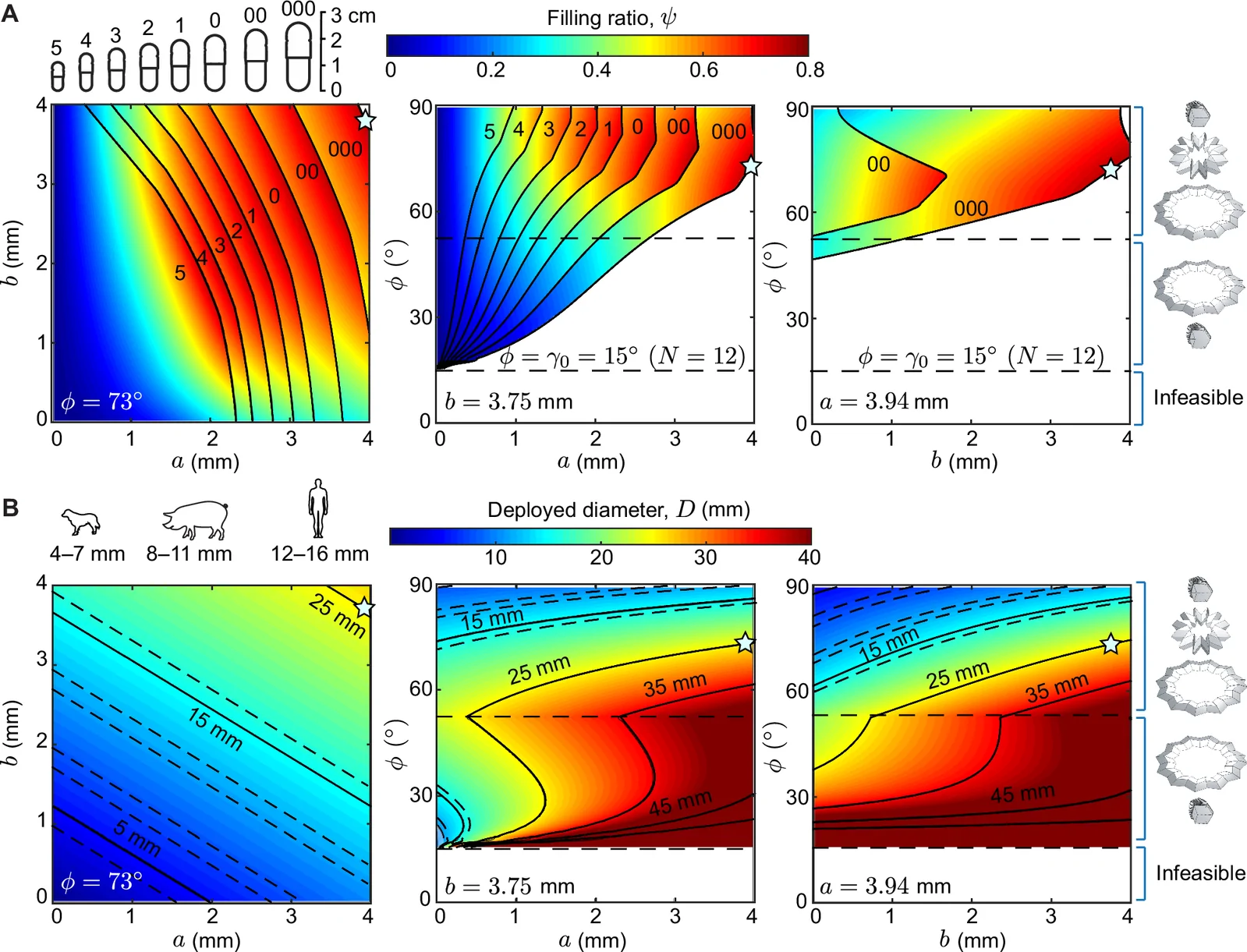
Characterization of shape-transformable metamaterial for encapsulation and deployment. **A** The effect of geometric parameters $a,{b}$, and φ on the capsule filling ratio, ψ, of the ODF while encapsulated in a standard ingestible capsule such as 000, 00, 0, 1, 2, 3, 4, 5. The contours represent the boundaries of the smallest ingestible capsule the system fits into. **B** The effect of geometric parameters on the ODF deployed diameter, D. The average range of pylorus diameter for dogs, pigs and humans are shown with dashed lines. The horizontal dashed lines in the second and third columns show the ranges of φ for which the device has three, two and no stable states. The star marker indicates the optimal ODF design prototyped and tested in animal models. The fixed value of the third parameter in each plot is selected from the optimized parameter set obtained by maximizing the capsule filling ratio while satisfying capsule size and pylorus diameter constraints.

As previously discussed, an orally-delivered ODF should fit inside an ingestible capsule for delivery and should become larger than the pylorus to reside in the stomach and prevent passage after delivery. To maximize the drug loading capacity and the amount of drug delivered to the body, the capsule filling ratio (ψ) of ODF should be maximized. The capsule filling ratio, ψ, is the ratio between the internal volume of the capsule being used for delivery and the volume of the ODF. The length of the ODF when encapsulated is linearly related to the thickness of the ODF sheets $(l=4{Nt})$ where N is the number of unit cells in the circular origami structure and t is the thickness of the sheet making the ODF. Therefore, it can be easily adjusted to fit the ODF inside any capsule. The capsule filling ratio, ψ, can be then defined as the ratio between the cross-sectional areas of the encapsulated ODF and that of the capsule (see Fig. 1D-left). We investigated the effect of geometric variables on ψ, the capsule filling ratio, and D, the deployed diameter, of the ODF, in Fig. 2A and 2B, respectively. Each graph shows the effect of two parameters:a and b (left), a and φ (middle), and b and φ (right) while the third parameter is fixed ($\varphi=73^\circ$, b=3.75 mm, and a=3.94 mm in the left, middle, and right plots) (see Optimization of the origami dosage form in the Supporting Information). We considered ${\gamma }_{0}=15^\circ$ (equivalent to N=12) to be constant across all plots (see the effect of ${\gamma }_{0}$ in Extra parameter study in the Supporting Information). The capsule filling ratio, as a function of the geometric parameters, is reported in Fig. 2A and is based on the size of the smallest standard ingestible capsule that the folded ODF fits into. We considered 000, 00, 0, 1, 2, 3, 4, 5 standard capsules and specified the range of geometric parameters of the ODFs that fit in that capsule with black solid lines/contours in Fig. 2A.

The color maps in Fig. 2B present the ODF outer diameter in the deployed configuration (D). The solid lines/contours correspond to D= 5, 15, 25, 35, and 45 mm, and the dashed lines represent the ranges of the pylorus sizes in human, swine, and dog. The white areas indicate ranges of parameters in the design space where the ODF cannot fit into any standard capsule or the design is geometrically impossible. The horizontal dashed lines in the color maps (middle and right) divide the φ design space to three sections with different possible configurations: the top section where the ODF shows all the three states shown in Fig. 1B; the middle section where only *state I* and *III* are possible; and the bottom section where the ODF is geometrically infeasible.

If φ fixed, increasing a or b results in higher values for both the capsule filling ratio and the deployed diameter of the ODF. Increasing φ while ${\gamma }_{0}$, b, and a are fixed, however, decreases both the capsule filling ratio and the deployed diameter. Thus, an increase in a and b positively impacts the optimization objectives while an increase in φ has a negative impact. We chose an ingestible 000-capsule, with an internal diameter of ${d}_{c}=9.15{{\rm{mm}}}$ and a length of ${l}_{c}=22.5{{\rm{mm}}}$ to deliver the ODF. Furthermore, given that the pylorus diameter in human is roughly 12–18 mm^45,46^, we fixed the ideal diameter of the ODF to be ${D}_{{{\rm{ideal}}}}=25{{\rm{mm}}}$, ensuring the ODF prolonged gastric residence. The optimum design of the ODF can be found using an optimization process as explained in Optimization of the origami dosage form of the Supporting Information. The results show that for a=3.94 mm, b=3.75 mm, ${\gamma }_{0}=14.3^\circ$, and $\phi=73^\circ$ the capsule filling ratio of the ODF maximizes at ψ=0.80. Since ${\gamma }_{0}$ determines the number of unit cells in each device ($N=180/{\gamma }_{0}$) we changed the ideal center angle to ${\gamma }_{0}=15^\circ$, which guarantees there are exactly N=12 unitcells in each ODF. For the optimized ODF design, the folding angle at the fully deployed configuration (*State II*) is ${\theta }_{d}=22^\circ$ and the limit value of the ODF’s radius, ${\rho }_{f}=11.35$ mm at *state III*. Knowing the geometry of the ODF, the thickness of the sheets used for its fabrication was chosen to be 0.4 mm, ensuring that the final ODF with its length of 19.2mmfits inside a 000-capsule of l=22.2 mm. The white asterisk insets on the graphs in Fig. 2 indicate the geometric parameters for the optimum ODF.

We note that the geometric design of the ODF primarily governs device deployment and gastric retention. In contrast, drug release is influenced indirectly by the geometry through its effect on the surface-area-to-volume ratio of the structure, which is largely determined by the thickness of the drug-loaded polymer sheets.

### Material formulation and in vitro characterization

The ODF was made of poly(ε-caprolactone) loaded with moxifloxacin as a model drug. Moxifloxacin is a fourth-generation fluoroquinolone antibiotic typically obtained as a crystalline powder (moxifloxacin hydrochloride). In pharmaceutical formulations, moxifloxacin particles are commonly present in the micron size range, with reported sizes typically spanning a few micrometers depending on processing conditions (e.g., 4–20 μm in microparticulate systems)^47^.

The crystalline nature of moxifloxacin is characterized by distinct diffraction peaks in X-ray diffraction (XRD) and well-defined morphological features under microscopy. When incorporated into polymeric matrices such as polycaprolactone (PCL), prior studies have shown that drug molecules can be uniformly dispersed within the polymer phase, forming a composite in which drug particles are embedded within the semi-crystalline PCL matrix^48^. In such systems, interactions between moxifloxacin and PCL are typically physical rather than chemical, as confirmed by Fourier Transform Infrared Spectroscopy (FTIR) and thermal analysis, with the drug remaining structurally stable while being distributed throughout the polymer network. The resulting composite often exhibits a heterogeneous microstructure, where dispersed drug domains contribute to mechanical weakening at higher loadings and facilitate diffusion-driven drug release.

In this study, we primarily used PCL with an average molecular weight of ~37 kDa (PCL37), which provides sufficient mechanical strength for prolonged gastric retention. Polycaprolactone (PCL) is a biodegradable and FDA-approved material known for its mechanical strength, elasticity, and ease of formation, making it suitable for fabrication of the orally administered forms^9,49^. Due to its low melting point ($70^\circ$ to $90^\circ$C), it is easy to incorporate drugs into PCL by mechanically blending the molten polymer and the drug powders. Scalable manufacturing processes, such as hot melt extrusion, can be used for making the mixture. We also evaluated the usage of lower molecular weight PCL (~25 kDa, PCL25), which resulted in reduced gastric retention time. This behavior is attributed to the lower mechanical strength and faster degradation rate of lower molecular weight PCL, which accelerates structural weakening and device passage.

**Fig. 3:**
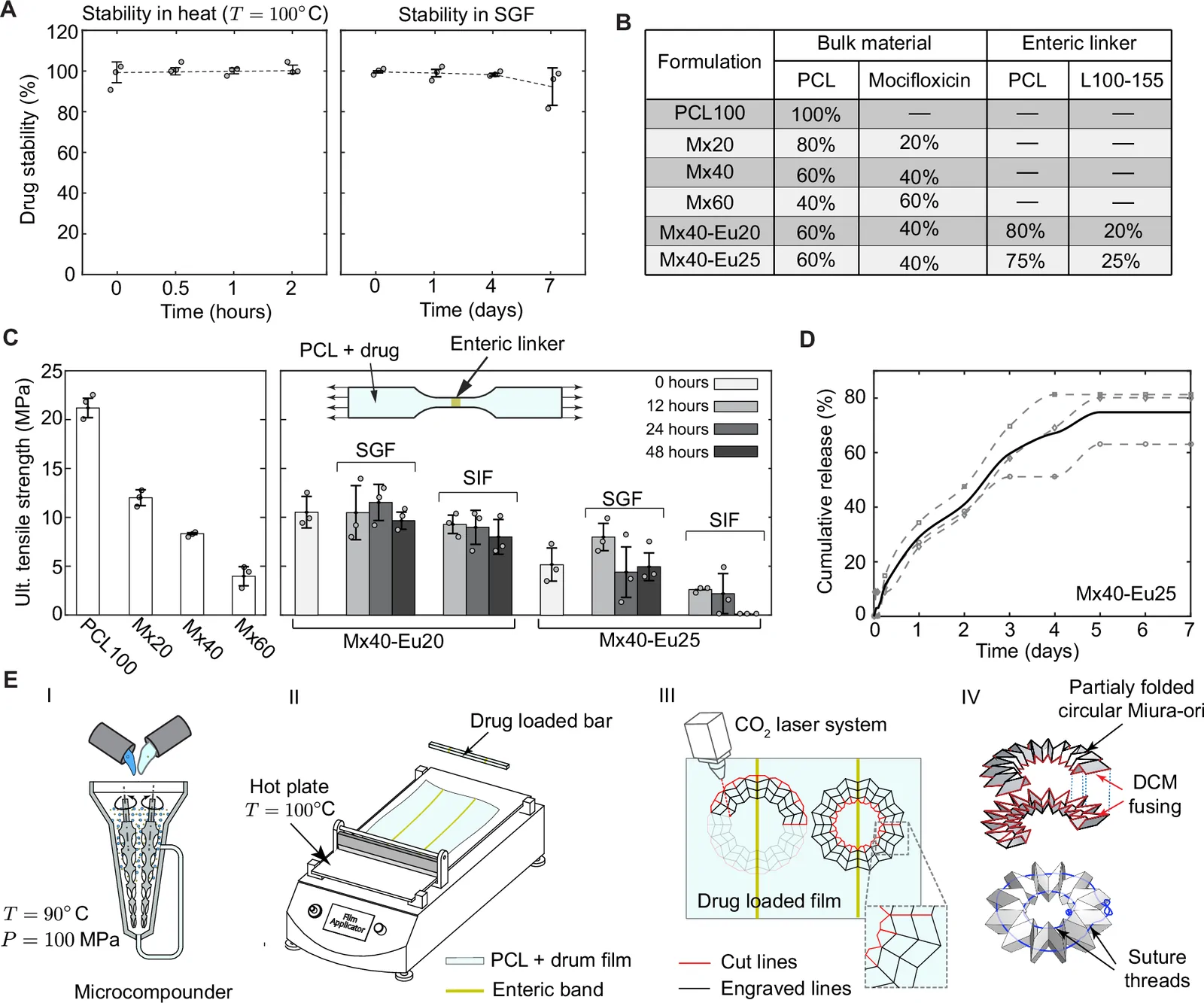
Material characterization and fabrication. **A** The stability of the sample drug, moxifloxacin, to a temperature $T=100^\circ$ C and exposure to simulated gastric fluid (SGF). The ODF fabrication involves three melting processes, requiring heating the mixture up to $100^\circ$ C for approximately an hour. Additionally, the ODF will be exposed to gastric fluid inside the stomach during its residency. **B** The list of candidate formulations for the fabrication of the ODFs’ bulk material and enteric linkers. **C** The candidate formulations are mechanically characterized by their ultimate tensile strength. The effect of added drugs on the mechanical strength of the PCL is shown at the left and the effect of SGF and SIF incubation at the right. **D** The in vitro drug release results show that around 70% of the total loaded drug is released from the ODF made of Mx40-Eu25 during five days of incubation in SGF. **E** A schematic of the fabrication process: 60% w/w PCL and 40% moxifloxacin (75% PCL and 25% Eudragit for the safety bands) were first mixed in a microcompounder. The drug-loaded mixture was cast into a bar with two safety bands incorporated at the middle of the bar. Thin films of drug-loaded PCL were made using a hot plate film applicator. The films were then cut into circular Miura-ori patterns using a laser cutter. Two patterns were finally fused using dichloromethane to make the toroidal shape origami dosage form. Quantitative measurements were performed using three independent samples (n=3). Data are presented as mean ± standard deviation, and error bars represent standard deviations.

As shown in Fig. 3A-left, the heat stability of moxifloxacin was verified by incubating the drug powder at $T=100^{{\rm{\circ }}}{{\rm{C}}}$ for two hours. Additionally, as seen in Fig. 3A-right, the stability in the harsh and highly acidic gastric environment (37°C, 100% humidity, and pH 1.5) was studied by loading the drug into PCL and incubating in simulated gastric fluid (SGF) for 7 days. In both cases, drug-loaded samples were incubated under the specified conditions for defined durations, after which the drug was extracted using a solvent and quantified using a stability-indicating high-performance liquid chromatography (HPLC) assay. These results show that moxifloxacin remains stable under both thermal processing and simulated gastric conditions.

Pure PCL is strong enough to easily tolerate the gastric contraction for weeks. However, as other substances are added to PCL, the sheets become more brittle which makes the fabrication process more challenging and the final ODFs more prone to failure. Adding drugs and including Eudragit safety bands in particular decrease the mechanical strength of the PCL films. For extended residency, it is critical that the ODF mechanically withstand the gastric contractions, requiring the dosage form to retain its mechanical strength during residency. In contrast, if the ODF passes the pylorus, the enteric films should break apart to ensure the secure passage of the device in the intestine. To find the right formulation for the ODF, we characterized the mechanical strength of different mixtures of drug and polymer with and without Eudragit safety bands (the list of formulations is shown in Fig. 3B) before and after incubation in SGF and simulated intestine fluid (SIF). As can be seen in Fig. 3C-left, adding the drug decreases the mechanical strength of the mixture. This is likely because the drug particles weaken the polymer network by reducing the number of bonds between the monomers. Specifically, we observed that when the drug concentration exceeded 40% of the total weight of the mixture, the sheets became weak and brittle, rendering them unsuitable for ODF fabrication. Therefore, we selected a 60% PCL and 40% w/w drug mixture to fabricate the ODFs.

The inclusion of the pH-sensitive safety bands, made of Eudragit L100-155^49^, decreased the mechanical strength of the thin films after incubation in both SGF and SIF, as shown in Fig. 3C-right. When the Eudragit component at the safety bands was at 25% w/w, the sheet could withstand SGF incubation without significant mechanical weakening, but it broke down in SIF within 48 hours. Therefore, we chose a 75% PCL and 25% Eudragit L-100155 mixture to fabricate the safety bands. Given these observations, we concluded that the ideal formulation for fabrication of the ODF is MX40-Eu25, which is a 60% PCL and 40% w/w moxifloxacin bulk, with a 75% PCL and 25% Eudragit safety band mixture. The mechanical characterization of the thin films was conducted by performing standard tensile tests of dog-bone samples of the films (see Mechanical characterization in the Supporting Information for more details).

When a PCL-made ODF was placed in gastric cavity, it swelled slightly and opened channels in the polymer for drug diffusion. To study the kinetics of the drug release, an in vitro study was performed using MX40-Eu25 sheets. As can be seen in Fig. 3D, the drug was released from the ODF to the SGF within five days, during which up to 70% of the drug was released at a near constant rate. The approximately linear release profile may be associated with the high surface-area-to-volume ratio and minimal thickness of the origami structure, which promote uniform exposure to gastric fluid. We note that the release behavior is primarily governed by diffusion through the polymer matrix and fluid-induced swelling, while the origami geometry modulates exposure area rather than fundamentally altering the underlying release mechanism.

The fabrication process of the ODFs is shown in Fig. 3E. First, PCL (60% w/w) was mixed with moxifloxacin (40% w/w) through melt mixing in a commercial twin screw micro-compounder (Xplore™ Instruments, Netherlands) at temperature $T=100^\circ$ C. The mixture was then molded into a thin sheet while small bands of PCL (75% w/w) and Eudragit L100-55 (25% w/w) were incorporated into the sheet using a film applicator (TQC, Netherlands). The thickness of the PCL-drug-Eudragit thin film was set at 0.4 mm. The pattern of the circular Miura-ori metamaterial was cut from the flat sheet using a laser cutter (VLS6.60, Universal Laser Systems, Inc., USA). The creases were cut as dashed lines with 50% spacing. Small flaps were added to the edges of the circular Miura-ori metamaterials to facilitate the gluing process. Two sheets of the circular origami were then fused to each other using dichloromethane (DCM) to make a tube. The ODF was left under a fume hood for 48 hours while the DCM evaporated. A surgical suture was then passed through the ODF to ensure its full closure while it was deployed inside the stomach (see Fabrication process in the Supporting Information for more details).

### In vivo gastric residence and extended drug release

**Fig. 4:**
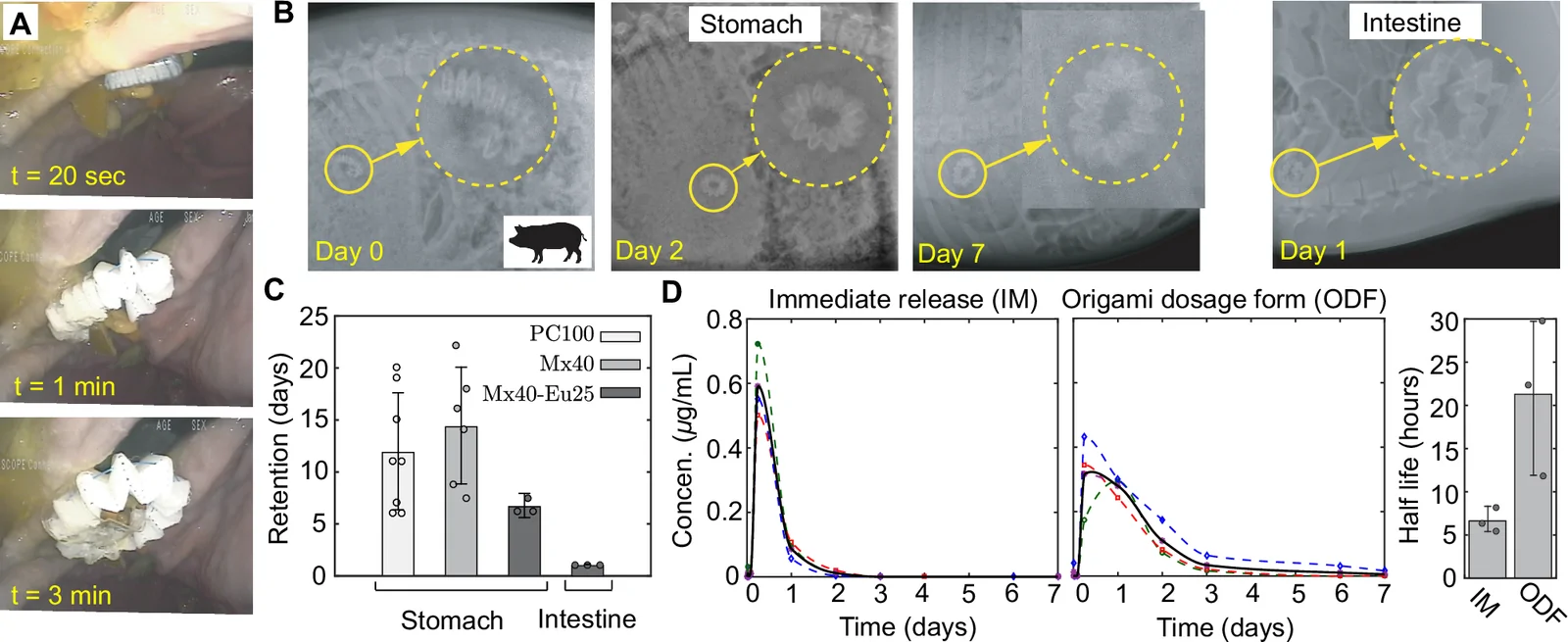
In vivo evaluation of gastric residence and drug release. **A** The snapshots showing capsule dissolution and deployment of a representative ODF in the stomach at different time points *t* = 20 s, 1 and 3 min taken by an endoscopic camera. **B** Abdominal radiographs (magnified view highlighted with a dashed yellow circle) obtained after the ODF administration. The images on the left represent the ODF-deployed configuration in the stomach at Day 0, 2, and 7 after delivery. The right image shows an ODF delivered directly into the intestine at Day 1. The magnified inset shows the segmented ODF in the intestine. The ODFs were loaded with 40% barium-sulfate to become radiopaque (see In vitro studies and Oral pharmacokinetics in the Supporting Information). **C** In vivo retention of the ODF deployed in the stomach and intestine of swine animal models (n≥3). The addition of moxifloxacin and the enteric linkers reduced the ODF retention period. The ODFs resided in the stomach for a minimum of one week while they passed after a day when deployed in the intestine. Data are presented as mean ± standard deviation. **D** In vivo plasma pharmacokinetics of immediate and sustained release of moxifloxacin (n=3). The concentration time profiles of immediate release (left) and sustained release by the ODF (middle) are presented. Each dosage form was tested in three animals, and plasma samples from each animal were processed three times. Data was first averaged within each animal (as shown by the dashed lines) and then between animals in each treatment group (as shown by the solid lines).

The ODF administration is shown in Fig. 4A. A loaded 000-capsule was placed inside the stomach of a swine via an endoscope and over tube under anesthesia (using tiletamine/zolazepam). Upon reaching the stomach, the ingestible capsule broke apart in approximately one minute, allowing the ODF to elastically recoil out. The ODF reached its deployed diameter in around three minutes (see Elastic release of circular Miura-ori metamaterial and movies included in S1 and S2 of the Supporting Information).

The gastric retention of the ODF was evaluated in vivo in Yorkshire swine (female, 50–120 kg). A total of 20 devices were administered during drug retention study, including 8 non-drug-loaded and 6 drug-loaded ODFs, delivered via nasogastric tube under short general anesthesia. Two preliminary retention studies using non-drug-loaded ODFs fabricated from PCL25 were conducted to compare PCL25 and PCL37 formulations. To verify the ODF’s retention, X-ray images were taken on a daily and bidaily basis, depending on the progression of the study, on anesthetized animals (see Fig. 4B). The non-drug ODFs were loaded with 40% w/w barium sulfate, a radio-opaque material, for X-ray visualization purposes. The drug-loaded ODFs, however, were made visible in X-ray with small metallic beads passed through their sutures. Moreover, to study the chance of intestinal blockage, six Mx40-Eu25 ODFs were administered three to the stomach and three to the intestine. X-ray images of the administered ODFs are shown in Fig. 4B. The ODFs stayed intact in the stomach for up to three weeks (with a minimum of a week) while their Eudragit safety bands broke apart in the intestine after only one day of administration. During the in vivo studies, the models maintained their normal diets and digestion. No signs of GI obstruction and discomfort, including vomiting, distention, or altered feeding behavior were observed in the animals.

The retention times of different ODFs are compared in Fig. 4C. All ODFs, with and without loaded drugs, stayed in the stomach for a minimum of 1 week, while all ODFs passed within one day after entering the intestine. Additionally, due to the drug diffusing into the stomach fluid, the drug-loaded devices (made of Mx40-Eu25) had a shorter retention time compared to the barium sulfate-loaded ODFs. The retention variability in Fig. 4C primarily reflects differences in gastric motility, pH environment, and GI geometry among individual animals rather than mechanical failure. It also demonstrates the effect of enteric linkers on ODF passage through the intestine. Our design successfully extends retention beyond the therapeutic window (4 days) in all cases. We note that drugs with different dissolution rates would affect the minimum retention time accordingly. Therefore, the design should be optimized for each drug candidate. We also note that the PCL sheets failure occurs gradually through drug dissolution and polymer degradation, leading to softening and crumbling rather than sharp fracturing. Once the device reaches the intestine, the neutral pH rapidly dissolves the enteric linkers, resulting in three soft segments that remain tethered by the suturing thread and pass safely. No sign of rigid or sharp fragments were observed  during our studies. Maintaining structural integrity beyond the drug-release window may be advantageous in ensuring consistent delivery during the therapeutic phase, while allowing sufficient time for safe degradation and passage thereafter. This behavior is consistent with a controlled degradation pathway that minimizes the risk of mechanical injury during gastrointestinal transit.

The oral pharmacokinetics of moxifloxacin, when dosed as an unformulated drug, are shown in Fig. 4D. An average maximum serum concentration (C_max_) of ∼0.6 *μ*g/mL was achieved 6 h post-dose. Drug levels declined to ∼15% of the average C_max_ on the first day, and after the second day, the drug was undetectable in the serum (Fig. 4D-left). In contrast, when dosed in the sustained release ODF (see Fig. 4D, right), the initial burst was significantly contained. Specifically, an average maximum drug concentration of ∼0.35 μg/mL was observed at 6 h post-dose. Most notably, serum concentration was ~0.245 μg/mL (~70% of average C_max_) on the first day, with drugs detected in the serum for up to four days. The therapeutic window of the drug is a function of the ODF’s geometry and fabrication, the drug-loading substance (PCL), the drug type, and the animal model biology. However, the rate of release, is a function of the ODF geometry, degradability and hydrophilicity of PCL, and the drug solubility. To increase the rate of drug release, the ODFs can be coated with biodegradable polymers such as poly(lactic-co-glycolic acid) or PLG, which controllably dissolve in gastric fluid. By precisely choosing the coating substance and controlling its thickness, the drug release rate can be controlled or even delayed in the body.

We conducted a non-compartmental pharmacokinetic analysis of our data and estimated the elimination rate constant of moxifloxacin dosed in an immediate release formulation and the sustained release ODF. When dosed in the sustained release ODF, the elimination rate constant of moxifloxacin was about 65% lower than that of the drug dosed in immediate release formulation (0.038 ± 0.018 versus 0.107 ± 0.021, *n* = 3; *p* < 0.05, conducted via a Student’s t-test).

## Discussion

We introduced an origami-inspired dosage form (ODF) for drug delivery into the stomach. The dosage form folds inside a 000-capsule for delivery. Upon capsule dissolution, the ODF deploys to a toroidal shape that does not pass the pylorus and can resist the stomach forces. The ODF shape and formulation are optimized to maximize the gastric residence of the ODF as well as the amount of drug it delivers. The ODF was used for multi-day delivery of moxifloxacin into the stomach of swine models. We demonstrated that the ODF can stay inside the stomach for up to three weeks. We also detected notable amount of the sample drug, moxifloxacin, in the animal models for up to four days.

None of the ODFs placed inside the animal models were captured after passing the GI tract. It is therefore not clear how the ODFs break apart when inside the stomach. However, it is expected that the ODF loses its mechanical strength due to drug diffusion and deforms under the stomach forces, which enables its passage through the pylorus. When in intestine, the enteric bands of the ODF broke apart and facilitate its safe passage through the rest of GI tract. This is supported by the in vitro data demonstrating the mechanical weakening of the formulations after SGF incubation and the dissolution of the enteric bands after reaching the intestine.

Considering its low melting point, PCL can be mechanically mixed with many drug powders, as long as the drug is stable at $< 100^\circ$ C, while PCL is in liquid state. Given that, the ODF proposed here can be used for prolonged delivery of different types of drugs and substances into the gastric cavity. Moreover, the single-material dosage forms are simple, relatively cheap to fabricate, and additionally have a low chance of GI tract blockage or other post-ingestion complications. Taken together, this makes ODFs a particularly promising candidate for low-cost prolonged drug delivery, especially for low-income regions of the world with limited access to medical professionals and emergency facilities.

Device failure is expected to occur through gradual material softening rather than brittle fracture. As drug diffuses out, the PCL matrix becomes increasingly compliant, and subsequent exposure to intestinal conditions accelerates linker dissolution and structural disassembly. This progressive degradation pathway reduces the likelihood of sharp fragment formation and supports safe passage through the gastrointestinal tract. However, in the unlikely event of obstruction, the device can be retrieved or fragmented endoscopically using standard tools.

The amount of drug can be loaded to the ODF is limited to <400 mg. The size of the ingestible capsule used for drug delivery mainly determines the size of the ODF and, consequently, the amount of drugs that can be loaded into it. The weight of the ODF that fits inside a 000-capsule is around 1 g; each ODF can therefore deliver around 400 mg of drug. Considering this, only low-dosage drugs are suitable for ODF delivery. Risperidone, used to treat mental disorders such as bipolar and schizophrenia, is a good candidate to be used for prolonged ODF delivery. To achieve higher doses, multiple ODFs could be administered simultaneously. Future work could focus on optimizing the origami-based folding design and film composition to enhance drug-loading capacity and adapt the system for a wider range of therapeutics. However, the origami-inspired ODF offers a promising platform that addresses the need for prolonged oral drug delivery systems introduced earlier in this study.

Potential applications of this platform include delivery of antibiotics, antiparasitics, and chronic medications where multi-day dosing is beneficial. By reducing dosing frequency, such systems may improve adherence and therapeutic outcomes, particularly in settings where consistent access to healthcare is limited. Such applications are particularly relevant for therapies where maintaining therapeutic levels over several days is critical, such as antibiotic regimens where missed doses can contribute to treatment failure or resistance.

This study has several limitations. First, device retrieval following intestinal passage was not feasible, limiting direct characterization of final failure modes. Second, in vivo studies were not designed to systematically evaluate the effect of geometric parameters on retention behavior. Third, comparisons with alternative sustained-release systems were not performed. Future work will address these aspects through expanded animal studies, imaging-based tracking, and comparative evaluations. Additionally, while in vitro and imaging data support the proposed disassembly mechanism, direct visualization of device breakdown within the gastrointestinal tract remains an important area for future investigation.

## Methods

ODF design, fabrication, in vitro characterization, and in vivo studies are described in the Results section and detailed further in the Supplementary Information. Statistical analyses were performed as described in the main text.

### Reporting summary

Further information on research design is available in the Nature Portfolio Reporting Summary linked to this article.

## Supplementary information

Supplementary Information

Description of Additional Supplementary Files

Movie S1.

Movie S2.

Supplementary Code 1

Reporting Summary

Transparent Peer Review file

## Source data

Source Data File

## Data Availability

The main data supporting the results in this study are available within the paper, Supplementary Information document and supplementary code and data package code-S1.zip. Source data are provided with this paper.

## Code availability

MATLAB script used for the statistical analyses and optimization, CAD files of the design, and the experimental data gathered during this study are available as Supplementary Material at cod-S1.zip.
