# Supplementary material for: An ingestible origami-inspired metamaterial for prolonged oral delivery of therapeutics
Source: Nat Commun. 2026 Jul 27;17:9090. doi: 10.1038/s41467-026-76028-y (PMC13518907; doi:10.1038/s41467-026-76028-y)
Supplement: Supplementary file 1 [file 41467_2026_76028_MOESM2_ESM.pdf]

<u>Description of additional Supplementary files</u>

Supplementary Movie 1: In vitro deployment of a circular Miura-ori metamaterial. The video shows the release of the folded structure in air, where the two ends rapidly expand and connect to form the fully deployed configuration (State II).

Supplementary Movie 2: In vivo deployment of the ODF. The video shows capsule dissolution and subsequent deployment of the origami device in the stomach of a swine model.

Supplementary Code 1- MATLAB codes used for optimization and the data collected during this study.
