# Supplementary material for: An ingestible origami-inspired metamaterial for prolonged oral delivery of therapeutics
Source: Nat Commun. 2026 Jul 27;17:9090. doi: 10.1038/s41467-026-76028-y (PMC13518907; doi:10.1038/s41467-026-76028-y)
Supplement: Supplementary file 3 [file 41467_2026_76028_MOESM7_ESM.pdf]

nature portfolio

Peer Review File

An ingestible origami-inspired metamaterial for prolonged oral delivery of therapeutics

Corresponding Author: Dr Giovanni Traverso

This file contains all reviewer reports in order by version, followed by all author rebuttals in order by version.

Version 0:

Reviewer comments:

Reviewer #1

(Remarks to the Author)
The authors present an "origami-inspired dosage form" (ODF) designed to extend the gastric residence time of orally administered therapeutics. The core innovation lies in a shape-shifting metamaterial based on a circular Miura-ori tessellation. This structure is composed of drug-loaded polycaprolactone (PCL) sheets that can be folded compactly into a standard 000-size gelatin capsule. Upon dissolution of the capsule in the stomach, the ODF deploys into a toroidal shape larger than the pyloric sphincter, preventing gastric emptying. The authors optimize the geometry for packing efficiency and deployment reliability, characterize the mechanical and drug-release properties of PCL-moxifloxacin composites, and incorporate pH-sensitive "safety bands" (Eudragit) to ensure safe passage upon reaching the intestine. In vivo studies in swine demonstrate gastric retention for up to three weeks and sustained release of moxifloxacin for up to four days with reduced burst release compared to immediate-release formulations.

While the work is interesting, there are several issues where the manuscript could be strengthened to meet the high standards of Nature Communications.

1.The paper claims "up to 40% weight loading capacity," but Fig. 3C shows a significant drop in mechanical strength at this loading. Is the ODF mechanically robust enough to survive gastric contractions at 40% loading for the full 3 weeks? The retention data for drug-loaded ODFs (Mx40) in Fig. 4C shows a wide variance (some passing at day 7, others day 21). The authors attribute this to drug diffusion weakening the polymer.
2.If the device relies on mechanical failure for clearance, the mode of failure matters. Does it fracture into sharp shards? Does it unfold? Does it crumble? Large, rigid fragments of PCL could pose a risk of intestinal perforation or obstruction, even if they pass the pylorus. The current "safety bands" (Eudragit) handle the intestinal transit scenario, but gastric clearance via mechanical fatigue is a separate, uncharacterized pathway.
3.High surface area aids drug release (pro) but increases drag forces during antral contractions (con), potentially leading to premature expulsion or blockage.Did the authors observe any signs of gastric outlet obstruction (e.g., vomiting, distention) in the pigs? How does food pass through or around the device? A discussion on the hydrodynamic permeability of the device compared to open-frame designs would strengthen the clinical contextualization.
4.Why is the therapeutic window (4 days) so much shorter than the physical retention time (7+ days)? This implies a "dead" device resides in the stomach for weeks after the drug is exhausted. This is suboptimal. Ideally, the device should degrade/pass shortly after drug depletion.

Reviewer #2

(Remarks to the Author)
The manuscript reports an ingestible, origami-inspired gastric-resident device constructed from drug-loaded polymeric sheets that fold into a capsule and deploy into an expanded configuration in the stomach. The authors aim to achieve multi-day gastric retention and sustained drug release, supported by benchtop characterization and limited in-vivo demonstrations. However, the novelty is vague and insufficiently justified, as similar expandable and deployable gastric-resident architectures have been extensively described in prior literature. The motivation for employing an origami-based metamaterial design is also not clearly articulated or experimentally substantiated. Overall, the results do not demonstrate a meaningful advancement over established systems, and the work lacks rigorous design characterization, mechanistic validation, and translational justification. Therefore, the manuscript is not suitable for consideration in Nat Comm.

Major Problems:
1. The expandable, capsule-delivered gastric-resident architecture has been thoroughly established in authors' prior work, including Kirtane et al. (Nat Commun 2018), Hayward et al. (Sci Rep 2018), and Bellinger et al. (Sci Transl Med 2016). These earlier studies demonstrate even more advanced drug-delivery performance and long-term retention. The introduction of an "origami metamaterial" does not appear to produce new functional benefits or improved performance. The manuscript should clearly articulate the motivation for this geometric approach and directly compare its advantages over prior systems, which is currently lacking.
2. The reported gastric retention is up to three weeks, whereas the drug loaded in PCL sheets is released within only ~4 days. This functional mismatch substantially weakens the motivation and translational relevance of the architecture. The authors should clarify the necessity of long-term retention if pharmacologically active release is completed within a few days.
3. Although the manuscript claims pH-responsive disassembly, the process is not mechanistically explained or experimentally validated. The authors acknowledge uncertainty in how and when the origami sheets break apart in vivo. Degradation studies, imaging, or endoscopic monitoring are essential to substantiate this claim.
4. Gastric retention critically depends on the structural integrity of the deployed architecture. The manuscript does not examine how partial or full disassembly affects retention, when failure occurs, or what conditions trigger loss of function. This is a major missing component.
5. The relationship between origami geometry and retention behavior is not studied. Key aspects such as retention failure mechanisms, retention duration as a function of geometry, safety implications, and drug-loading capacity should be systematically evaluated.
6. The animal study appears to be a single illustrative demonstration rather than a systematic investigation. Important factors —including the effect of feeding, gastric motility, mechanical stresses, structural robustness, and cyclic degradation—are not evaluated. These are required to justify functional reliability.
7. The manuscript does not convincingly articulate a clinical scenario where the proposed architecture offers advantages over existing long-acting oral delivery systems or over simpler, well-studied gastric-resident technologies.
8. The claim that origami-based architecture induces slow or near-zero-order release is not adequately compared to other mature approaches (e.g., drug-loaded hydrogels, foams, polymer matrices). Without comparative data, the relevance of the origami structure for drug-release control remains unclear.
Minor Problems:
1. Figure 2: Please clarify the rationale for selecting the specific fixed value of the third parameter (a, b, or ϕ) in each analysis.
2. Figure 3A: Please define what is meant by "drug stability" in this context and describe the experimental methodology used to quantify it.
3. Line 81: The statement "This innovation has the potential to benefit large populations worldwide…" is overstated and lacks supporting references. Please also clarify what "treatment adherence" specifically refers to in this context.
4. Line 244: The term "safety elements" is introduced without prior definition, which may confuse readers. Although it is explained later in the manuscript, a brief definition should be provided at first mention to improve readability and clarity for the audience.
5. Line 253: More detailed information on the selected drug and its characterization is needed, including particle size, microscopy, and characterization of the PCL–drug composite.
6. Line 270: Please clarify and provide evidence for the claim that minimal thickness and high surface area yield near-zero-order drug release.
7. Line 294: "Several animals" should be specified explicitly (n = ?).
8. Line 306: This claim is unsupported by experimental evidence; additional validation is needed.
9. Figure 4B: The Day-1 intestinal image is difficult to interpret. Labels, scale bars, and schematic orientation are required. It is also unclear why the device remains circular and how its size relates to the intestinal lumen.

Reviewer #3

(Remarks to the Author)
This manuscript describes an orally ingestible origami-based drug delivery device intended for prolonged gastric residence. The authors justify the folding design using a kinematic model and further demonstrate the concept through in vitro and in vivo experiments (swine model). Moxifloxacin was used as the model drug. The use of a foldable geometry to enable pylorus-no-passing deployment, combined with the adoption of biodegradable materials, is interesting.

The geometric design is well-motivated, particularly in terms of capsule-size compatibility, maximization of the filling ratio, and maximization of the deployed diameter. Material characterization, including environmental stability, drug loading stability, and drug release behavior, is also well studied. However, throughout the manuscript, the extent to which the origami architecture contributes uniquely to the drug release behavior remains unclear, both as the selection of the methodology and as its outcomes. At present, the design feels somewhat "origami-first", and alternative structural strategies are not discussed, if any. Comparative in vitro studies could strengthen the argument.

Regarding the deployment mechanics, I understand that State II and State III correspond to the behavior in both the single-layer and double-layer configurations. And when the structure is configured as a double-layer metamaterial, the transition from State II to State I is inhibited (due to collision between edges). If collision did not occur, e.g. passing each other, would a fully deployed configuration be reachable? As currently presented, the plots of γ and ρ in Fig.1 do not intuitively communicate the underlying mechanism. Revising the visual explanation, e.g. by adding an intermediate deployment photograph, would improve some clarity. The conceptual image in Fig.1C doesn't describe the entire scenario (e.g. drug release). The scaling in Fig.1E (right), while it looks different from the left image.

Movie S1 gives me an impression that θ minimally changes during deployment and that the structure behaves similarly to a linear spring. Since one of the stated motivations for using origami is the high surface-area-to-volume ratio, it would be valuable to include additional quantitative comparison across different θ conditions. Optionally, a comparison of devices with different geometric parameters in in-vivo experiments (e.g., in Fig.4D) may further strengthen the justification (even a comparison with a planar sheet).

Fig.S1 also requires improvement. The unit-cell transition from left to right in panel B is not sufficiently clear, and the relationships between State I, State II, and (unshown) State III are difficult to interpret, especially when they look different when with the meta-material configuration. Important parameters, such as ρf ≈ D/2, are missing from the figure. While Fig.1B illustrates the transformation sequence, key parameters are omitted, making the associated plot difficult to interpret.

The Supplementary Information appears somewhat rough. There are repeated equation numbers (e.g., 3 and 4), some equations lack numbering, and several variables (such as filling ratio) are not defined until late in the SI despite appearing repeatedly in the main text. A more top-down description of the design logic, explaining which parameters drive which behaviors, even without equations, would help the reader. Also, is the notation for the double-overlined variable b explicitly defined?

In Fig.1, it would be helpful to indicate the θ range used in the actual device. The repeated reference to high surface-area-to-volume ratio is not well supported; currently the justification remains at the level of “the structure contains pleats”.

The in vivo study is well conducted; however, rather than simply stating that the experiment was performed, it would be helpful to articulate what differences were expected compared to in vitro conditions (e.g., pressure, motility) and whether the data confirmed or contradicted those expectations.

Conceptually related prior work, including Miyashita et al., Ingestible, Controllable, and Degradable Origami Robot for Patching Stomach Wounds (2016), IEEE ICRA, or the corresponding patent US10470799B2, is not cited. The recent work by Yuan et al., Magnetic-Guided Flexible Origami Robot toward Long-Term Phototherapy of H. pylori in the Stomach would also be relevant.

Finally, as also noted in Ref 1, it would be useful to discuss potential medical intervention if the deployed device obstructs gastric flow. Supplemental video 1 shows that the gelatin capsule was not fully degraded even after three minutes; this appears inconsistent with the text and may require clarification. Since the device appears to release substantial elastic energy upon deployment, discussion of possible risks during esophageal transit and capsule mechanical robustness would be valuable.

Minor comments:
- In Video S1, the recording stops immediately after deployment. An additional 30 seconds to 1 minute of footage following deployment would be helpful.
- In Fig.2B, using different dashed line styles to represent the pylorus diameter of different animals might be better.
- The x-axis label of Fig.3A left does not match the description in line 239. Is it two days or two hours?
- In line 302, the ODF stays intact in the stomach for up to three weeks, since the drug release experiment shows the drug will be undetectable in a week. Is this testing on purpose, or does it just show the ODF is just stuck in the stomach even if the drug-releasing task has completed?
- In Fig.4D, it would be good to show the meaning of the different dashed lines.
- In line 367, why “the amount of drug that can be loaded to the ODF is limited to <400mg?”

Version 1:

Reviewer comments:

Reviewer #1

(Remarks to the Author)
The authors have comprehensively addressed all of my previous concerns. The revisions have significantly improved the clarity and quality of the manuscript. I am now pleased to recommend the paper for publication in Nature Communications.

Reviewer #2

(Remarks to the Author)
The authors have addressed my comments.

Reviewer #3

(Remarks to the Author)
Although the response letter does not explicitly indicate all locations of the implemented revisions, the authors have sufficiently addressed my initial concerns, and the corresponding changes are implemented both in the main manuscript and the Supplementary Information appropriately.

Open Access This Peer Review File is licensed under a Creative Commons Attribution 4.0 International License, which permits use, sharing, adaptation, distribution and reproduction in any medium or format, as long as you give appropriate credit to the original author(s) and the source, provide a link to the Creative Commons license, and indicate if changes were made.
In cases where reviewers are anonymous, credit should be given to 'Anonymous Referee' and the source.
The images or other third party material in this Peer Review File are included in the article's Creative Commons license, unless indicated otherwise in a credit line to the material. If material is not included in the article's Creative Commons license and your intended use is not permitted by statutory regulation or exceeds the permitted use, you will need to obtain permission directly from the copyright holder.
To view a copy of this license, visit https://creativecommons.org/licenses/by/4.0/
