# Supplementary material for: An ingestible origami-inspired metamaterial for prolonged oral delivery of therapeutics
Source: Nat Commun. 2026 Jul 27;17:9090. doi: 10.1038/s41467-026-76028-y (PMC13518907; doi:10.1038/s41467-026-76028-y)
Supplement: Supplementary file 4 [file 41467_2026_76028_MOESM1_ESM.docx]

Supplementary Information for

**An ingestible origami-inspired metamaterial for prolonged oral delivery of therapeutics**

Farhad Javid^1^, Sahab Babaee^1,2,3^, Jasmine Quigley^1^, Ameya Kirtane^1,3^, Hormoz Mazdiyasni ^1,4^, Jaimie Rogner^1^, Cody Cleveland^1^, Taylor Bensel^1^, Vance Soares^1^, Joy E. Collins^1^, Kaithlyn Hess^1^, Shane McDonnell^1^, Alison M. Hayward^1,5^, Robert Langer^1,4^*, Giovanni Traverso^1,2,3^*

^1^Department of Chemical Engineering and Koch Institute for Integrative Cancer Research, Massachusetts Institute of Technology, Cambridge, MA 02139, USA.

^2^Department of Mechanical Engineering, Massachusetts Institute of Technology, Cambridge, MA 02139, USA.

^3^Division of Gastroenterology, Brigham and Women’s Hospital, Harvard Medical School, Boston, MA 02115, USA.

^4^Institute for Medical Engineering and Science, Massachusetts Institute of Technology, Cambridge, MA 02139, USA.

^5^Division of Comparative Medicine, Massachusetts Institute of Technology, Cambridge, MA 02139, USA.

*Corresponding authors. E-mail: rlanger@mit.edu (R.L.); cg20@mit.edu (G.T.)

**This file includes:**

**Fig. S1.** A) Planar and B) circular Miura-ori patterns; undeformed and deformed unit cells and origami patterns are shown.

**Fig. S2.** The effect of central angle $\gamma$ on the capsule filling ratio is shown for $\gamma=22.5^{\circ}(N=8), 18^{\circ}(N=10)\mathrm{and}15^{\circ}(N=12)$.

**Fig. S3.** Fabrication process; A) and B**)** poly(ε-caprolactone) and the sample drug, moxifloxacin are mixed in a commercial mixture. C) the mixture is molded into a bar while bands of Eudragit L100-150 are added to the bar. D) The bar is formed into a flat sheet with 0.24-0.4 mm thickness. E) The structure patterns are cut from the sheets. F) small flaps are added to the pattern to facilitate the fusing process. G) Two layers are fused using dichloromethane (DCM) and a surgical suture is passed through the device to ensure its closure. H and I) The final device inside and out of a 000 capsule is shown.

**Fig. S4.** In vitro deployment of the ODF; snap-shots of the device taken from a high-speed camera recording during ODF opening.

**Fig. S5.** Mechanical characterization; A) The dogbone samples and the testing apparatus are shown at left and some strain-stress curves obtained during the testing of the dogbone samples are shown at right. B) The funnel and normal compression tests performed on a drug-loaded ODF.

**Movie S1.** Recoiling; in vitro release of a circular origami tube.

**Movie S2.** Deployment; in vivo release of the origami dosage form (ODF) into the pig stomach.

**Code S1.** MATLAB codes used for optimization and the data collected during this study.

**Notation summary:**
$a,b$: unit cell edge lengths
$\phi$: angle between unit cell edges
$\theta$: folding angle
$\theta_{d}$: folding angle at deployed state (*State II*)
$\rho$: outer radius of unit cell
$D$: deployed diameter of the ODF
$N$: number of unit cells
$\psi$: capsule filling ratio

**Supplementary Notes**

**Miura-ori fold patterns**

The planar and the circular Miura-ori fold patterns are shown in **Fig. S1**. As can be seen, the parallel creases in the planar Miura-ori are replaced with inclined radial ones. The facets of the circular Miura-ori are then aligned around concentric circles. To form a 3D torus, only two rows of facets (one row of unit cells) in the radial direction are required. The unit cells in circular Miura-ori are hence stacked in a row to fill the circumference of a circle. If a full turn ($2\pi$ rad) is divisible with the central angle of the unit cells ($\gamma$), an exact number of the unit cells can be fit in a full circumference. The 3D toroidal Miura-ori metamaterial is made by fusing two circular patterns, each consisting of a single row of unit cells, arranged around a full circle.

**Fig. S1**. Miura-ori patterns; A) Planar and B) circular Miura-ori origamis. Undeformed, deformed, and folded unit cells and metamaterials are shown.

**Geometric characterization of circular Miura-ori origami**

The undeformed (*state I*) and fully-folded (*state III*) configurations of the origami-inspired metamaterial are characterized by ($\theta, \gamma, \rho$) $=$ ($\pi, \gamma_{0}, \rho_{0}$) and ($0, 0, \rho_{f}$), respectively. The Miura-ori pattern becomes flat in $xz$ plane when it is fully folded ($\theta=0$). The outer radius of circular Miura-ori, $\rho_{f}$, is hence degenerate. Here, $\rho_{f}$ is defined in the limit as $\rho\left. \right|_{\theta\to0^{+}}$. The geometry of the metamaterial is designed so that, through the course of its deformation, the structure experiences a stable *state II* defined by ($\theta_{d}, \gamma_{0}, D/2$), where $\theta_{d}$ is the deployed fold angle and $D/2=\rho_{0}$, $D$ is the outer diameter of the metamaterial at the deployed configuration (see the graphs on **Fig. 1B-right**). In this design, $\rho$ decreases monotonically as $\theta$ changes from $\pi$ to $0$, however, $\gamma$ increases as the folding starts, resulting in overlapping the open ends of the origami (see the second inset on **Fig. 1B-left**). As the folding angle, $\theta$, decreases further, $\gamma$ decreases and reaches $its$ initial value $\gamma_{0}$ where a full-circle origami is created (*state II* in **Fig. 1B**). At lower folding angles $\theta>\theta_{d}$, $\rho$ remains almost unaltered but $\gamma$ sharply decreases to 0 yielding the structure to approach the fully-flat configuration (*state III* in **Fig. 1B**).

Using simple trigonometric relations, we can show that,

$$\gamma=\phi_{d}-\lambda_{d}$$

(1)

$$\rho=\frac{\sin\phi}{\sin\gamma}\left( b+2a\frac{\sin\gamma_{0}}{\sin\left( \phi-\gamma_{0} \right)} \right).$$

(2)

Here, $\phi_{d}$ and $\lambda_{d}$, depicted in **Fig. S1B** for the deformed unit cell, correspond to the projected angles of the undeformed geometry shown in **Fig. 1A** and **Fig. S1B** in the planar coordinate system. Now, considering that the parallel lines remain parallel through deformation, we can show that(Gattas & You, 2014),

$$\cos2\phi_{d}=\frac{\sin^{2} \phi\cos\theta+3\cos^{2} \phi-1}{1-\sin^{2} \phi\cos\theta+\cos^{2} \phi} ,$$

(3)

and

$$\cos2\lambda_{d}=\frac{\sin^{2} (\phi-\gamma)\cos\theta+3\cos^{2} (\phi-\gamma)-1}{1-\sin^{2} (\phi-\gamma)\cos\theta+\cos^{2} (\phi-\gamma)}.$$

(4)

The folding angle, $\theta$, changes from $180^{\circ}$ to $0^{\circ}$ during the folding process. The *state II* shown in **Fig. 1** emerges during folding if $\gamma(\theta)$ increases right after folding starts, that is,

$$\frac{d\gamma}{d\theta}|_{\theta\to{180^{\circ}}_{-}}>0$$

(5)

where $\theta\to{180^{\circ}}_{-}$ means $\theta$ approaching $180^{\circ}$ while $\theta<180^{\circ}$. By replacing eqs. (2) and (3) in eq. (1) and taking the derivatives, after some simplifications we can show that eq. (5) results in

$$\sin2\phi>\sin2\left( \phi-\gamma_{0} \right).$$

(6)

If $\sin2\varphi>\sin2(\varphi-\gamma_{0})$, *stage II* is geometrically possible when the ODF deforms. In this case, $\rho_{f}$ is determined by evaluation of eq. (2) at $\theta\to0$. Here, $\theta_{d}$ is found by solving eq. (1) for $\gamma=\gamma_{0}$, and $D$ is determined by evaluating eq. (2) at $\theta_{d}$. These geometric relations provide the analytical basis for the optimization framework used in the main manuscript (Fig. 2) to identify the optimal ODF design parameters.

**Optimization of the origami dosage form**

The geometric design of the ODF can be defined as an optimization problem as,

| $\max_{[a, b,\gamma_{0}, \phi]} \psi(a, b,\gamma_{0}, \phi)$ | (7) |
| --- | --- |

Subject to

$$\left\{ \begin{aligned} D=D_{\mathrm{ideal}} \\ d=d_{c} \\ \sin2\phi>\sin2(\phi-\gamma_{0}) \end{aligned} \right.$$

(8)

where $\psi$ is the capsule filling ratio defined in the manuscript. The equality constraints guarantee that the ODF has an ideal diameter of $D_{\mathrm{ideal}}$ when it opens and fits inside an ingestible capsule of $d_{c}$ when folded. The inequality constraint is added to make sure that the optimum design has a stable *state II*.

We use genetic algorithm module in MATLAB R2017 (Mathworks Inc.) to find that $a=3.94$ mm, $b=3.75$ mm, $\gamma_{0}=14.3^{\circ}$, and $\phi=73^{\circ}$ maximizes the capsule filling ratio of the ODF to $\psi=0.80$. Since $\gamma_{0}$ determines the number of unit cells in each device ($N=180/\gamma_{0}$) we changed the ideal center angle to $\gamma_{0}=15^{\circ}$ which ensures there are exactly $N=12$ unit cells in each ODF. Knowing the geometry of the ODF, the thickness of the sheets used for its fabrication is chosen to be 0.4 mm so that the final ODF of 19.2mm long fits inside a 000-capsule of $l=22.2$ mm. The stars on the graphs in **Fig. 2** show the geometric parameters for the optimum ODF. For this design, the folding angle at the fully deployed configuration (*State II*) is $\theta_{d}=22^{\circ}$ and the outer radius when fully folded (*state III*) is $\rho_{f}=11.35$ mm.

**Extra parameter study**

The effect of central angle $\gamma$ (related to the number of unit cells, $N$, in a circular Miura-ori metamaterial by $N=\frac{\pi}{\gamma}$) on the capsule filling ratio of the origami dosage form (ODF) is shown in **Fig. S2**.

**Fig. S2**. Parametric study: the effect of central angle $\gamma$ on the capsule filling ratio is shown for $\gamma=22.5^{\circ}(N=8), 18^{\circ}(N=10)\mathrm{and}15^{\circ}(N=12)$.

As the number of unit cells around the circumference increases ($\gamma$ decreases), the maximum capsule filling ratio increases for each standard capsule. However, increasing the number of unit cells makes the ODF softer and hence may lead to shorter retention time in stomach.

**Fabrication process**

The fabrication process of the origami-inspired dosage form is shown in **Fig. S3**. The PCL and the drug are first mechanically mixed (**Fig. S3A**) and then melt mixed in a microcompounder system at 90-100° C (**Fig. S3B**). The mixture is then casted into a small rectangular bar using a silicon mold (**Fig. S3C**). The bar is then used in a film applicator to make a thin sheet of drug-loaded polymer (**Fig. S3D**). The pattern of the circular Miura-ori is cut from the drug-loaded thin sheets using a laser cutter; while the border of the pattern are cut with 100 pulse per inch (PPI) the creases of the pattern are marked with 10 PPI (**Fig. S3E**). For the ease of fusing, small flaps are added to the inner and outer edges of the layers (**Fig. S3F**). Two layers of patterns are fused to each other, with the help of extra flaps, using dichloromethane (DCM) and left in a fume hood for 48 h to allow complete evaporation of dichloromethane (DCM) (**Fig. S3G**). A suture is passed through the ODF to make sure it gets a circular shape after deployment inside the stomach (**Fig. S3H**). Tightening the suture thread could also generate compressive pretention in the ODF making it mechanically more stable. Such device can be fitted inside a 000 ingestible capsule for delivery (**Fig. S3I**). The observed deformation and failure behavior is consistent with the gradual softening mechanism described in the main manuscript, supporting a non-fracturing mode of device degradation.

Fig. S3. Fabrication process; A) and B) poly(ε-caprolactone) and the sample drug, moxifloxacin are mixed in a commercial mixture. C) the mixture is molded into a bar while bands of Eudragit L100-150 are added to the bar. D) The bar is formed into a flat sheet with 0.24-0.4 mm thickness. E) The structure patterns are cut from the sheets. F) small flaps are added to the pattern to facilitate the fusing process. G) Two layers are fused using dichloromethane (DCM) and a surgical suture is passed through the device to ensure its closure. H and I) The final device inside and out of a 000 capsule is shown.

**Elastic release of circular Miura-ori metamaterial**

To show the recoiling of the circular Miura-ori to state II, we have studied its in vitro release from a fully folded configuration (*state III*) in air. To this end, one end of the ODF is glued to a fixture to keep it in place while its other end is folded and contained using small surgical pliers. The spring-back of the metamaterial is shown in **Fig. 4** in six snapshots after the release. During the spring-back, the ODF behaves similar to a linear spring released from a pre-compression. Due to the release of potential energy of the folded ODF, its folding angle, $\theta$, changes from 0 to slightly higher than $\gamma_{0}$, the designs’ central angle. The highest angle we have measured during the elastic release of the ODF is measured on the top-right corner of Fig. S4 as $\theta={22}^{\circ}$. As can be seen here, the device opens up quickly (~ 30 ms) in air while the suture thread is removed from it. An slow-motion movie of this experiment is shown in Movie S1.

Fig. S4. In vitro deployment of the ODF; snap-shots of the device taken from a high-speed camera recording during ODF opening.

**Mechanical Characterization**

The mechanical characterization of the materials was performed on standard dog bone samples cut from the same thin films used for the device fabrication. The experiments were performed on a Universal testing machine (Instron Inc.). All tests were repeated at least three times. For evaluating the effect of the Eudragit safety bands, the bands were embedded exactly at the center of the dog bone samples. Ruptures in these cases occurred at or near the safety elements. The dog-bone samples, the testing apparatus and some strain-stress curves obtained during the experiments are shown in **Fig. S5A**.

To assess the ability of the ODF to resist to resist the forces of the migrating motor complex, the mechanical stiffness of the drug-loaded ODF is experimentally characterized. Considering the topology and mechanical properties of the stomach wall, the device can deform in lateral and axial directions due to the stomach contractions. Hence, as shown in **Fig. S5B**, a customized funnel and normal compression tests are performed on the device. The force required to fold the ODF inside the funnel or under compression is significantly higher than 3 N, which is commonly reported as the upper range of gastric contraction forces. The observed deformation and force response are consistent with the non-brittle failure behavior described in the main manuscript, supporting a gradual softening mechanism during in vivo degradation.

**Fig. S5.** Mechanical characterization; A) The dog-bone samples and the testing apparatus are shown at left and some strain-stress curves obtained during the testing of the dog-bone samples are shown at right. B) The funnel and normal compression tests performed on a drug-loaded ODF.

**In vitro studies**

The rate of drug release from thin MX40-Eu25 sheets used to fabricate the ODF was measured in vitro. For this study, sheets of defined surface area were placed in simulated gastric fluid (SGF, pH 1.2) in an incubator shaker (New Brunswick™, Innova®44, Eppendorf, NY, USA) at $37^{\circ}$ C and 150 rpm. At predefined time points, 1 mL of the release medium was collected and stored at −20 °C for analysis, and replaced with fresh SGF. Drug release was monitored for up to seven days. The concentration of moxifloxacin in the release medium was quantified using high-performance liquid chromatography (HPLC). HPLC was performed on an Agilent 1260 Infinity HPLC system (Agilent Technologies Inc., USA). Five microliters of the medium were injected onto a Poroshell 120 EC-C18 column (4.6 × 50 mm, 2.7 µm) held at 50◦ C. The organic and aqueous phases were acetonitrile and potassium dihydrogen orthophosphate buffer (0.136% potassium dihydrogen orthophosphate, adjusted to pH-3 using phosphoric acid) respectively. The solvents were delivered at a flow rate of $1 \mathrm{mL}/\min$ at 1 mL/min, starting at a composition of 0% aqueous phase at time zero changing to 100% aqueous phase over 2.5 minutes. The run time was five minutes, followed by a three minutes of post time. Moxifloxacin was detected using a diode array detector at a wavelength of 293 nm.

**Oral pharmacokinetics**

All animal protocols were approved by the Committee on Animal Care at Massachusetts Institute of Technology. To determine the oral pharmacokinetics of moxifloxacin delivered using the ODF, we treated each female Yorkshire pig with three ODFs loaded with 40% moxifloxacin placed in gelatin capsules as described before. At various times following dosing, the pigs were sedated, and 1−2 mL of blood was collected from the mammary vein. The blood was transferred to a gold top tube (Biolegend Vacutainer, Becton Dickinson and Co., NJ, USA), and serum was separated by centrifugation at 1800g, 10 min at 4◦C. Drug concentrations in the serum was analyzed using an ultra-performance liquid chromatography coupled with mass spectrometry (UPLC-MS/MS). UPLC separation was carried out on a Waters UPLC aligned with a Waters Xevo TQ-SMS mass spectrometer (Waters Ltd.). MassLynx 4.1 software was used for data acquisition and analysis. Chromatographic separation was performed on an Acquity UPLC Charged Surface Hybrid C18 (50×2.1 mm; particle size, 1.7 µm) at 50◦ C. The mobile phase consisted of acetonitrile with 0.1% formic acid, and a 10 mM ammonium formate solution. The mobile phase was forced at a rate of 0.6 mL/min using a solvent gradient composition. The initial gradient (100%) was held for one minute and then was lowered to 50% and 20%, respectively, over the next 15 and 30 seconds. The gradient was finally brought to 0% over the next minute and was held for additional 30 seconds. At 0.25 min, the gradient was shifted back to 100% and held constant until the end of the run for column equilibration. The total run time was 4.0 min, and the sample injection volume was 2.5µL. The mass spectrometer was operated in the multiple reaction monitoring mode. The sample introduction and ionization were performed using electron spray ionization (ESI) in the positive-ion mode.

Stock solutions of moxifloxacin and an internal standard ciprofloxacin were prepared in methanol at a concentration of 500 µg/ml. A nine-point calibration curve ranging from 2.5 to 1000 ng/ml was prepared. Quality control samples were prepared in a similar procedure using an independent stock solution at three concentrations (2.5, 25, and 250 ng/mL). Two hundred micro- liters of internal standard was added to 100 $\mu L$ of sample solution to cause precipitation. Samples were vortexed and sonicated for 10 min and then placed in a centrifuge for 10 min. Two hundred microliters of solution was pipetted into a 96-well plate containing 200 mL of water. Finally, 2.5 µL of sample was injected into the UPLC-ESI-MS system for analysis.

**Movie S1. In vitro deployment of a circular Miura-ori metamaterial.** The video shows the release of the folded structure in air, where the two ends rapidly expand and connect to form the fully deployed configuration (*State II*).

**Movie S2. In vivo deployment of the ODF.** The video shows capsule dissolution and subsequent deployment of the origami device in the stomach of a swine model.

**Code S1. Codes and data.** MATLAB codes used for design and optimization of the device geometry, the CAD files and the data collected during this study.

All supplementary analyses presented here directly support the mechanical, geometric, and drug-release behaviors described in the main manuscript.

**References**

Gattas, J. M., & You, Z. (2014). Miura-Base Rigid Origami: Parametrizations of Curved-Crease Geometries. *Journal of Mechanical Design, Transactions of the ASME*, *136*(12), 1–10. https://doi.org/10.1115/1.4028532
