# Supplementary material for: An ingestible origami-inspired metamaterial for prolonged oral delivery of therapeutics
Source: Nat Commun. 2026 Jul 27;17:9090. doi: 10.1038/s41467-026-76028-y (PMC13518907; doi:10.1038/s41467-026-76028-y)
Supplement: Supplementary file 5 [file 41467_2026_76028_MOESM5_ESM.zip › Code-S1/fig3/L100-55_02_10_2017/DogBone_SIF_2_1.is_tens.pdf]

Quasi-static tensile test to failure.

## Specimen 1 to 1

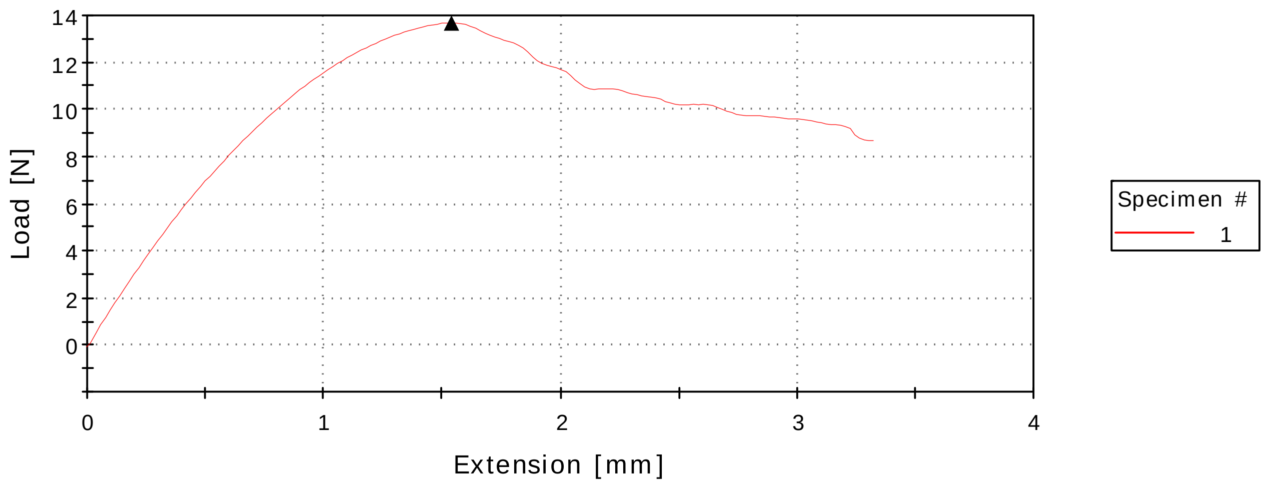


| | Rate 1 [mm/min] | Time at Tensile strength [s] | Extension at Tensile strength [mm] | Load at Tensile strength [N] |
|---|---|---|---|---|
| 1 | 12.00000 | 7.70000 | 1.53998 | 13.68612 |

| | Tensile strain (Extension) at Tensile strength [mm/mm] | Tensile stress at Tensile strength [MPa] | Poisson's ratio (Chord) | Modulus (Automatic) [MPa] |
|---|---|---|---|---|
| 1 | 0.03949 | 1.36861 | ----- | 58.22283 |

| | Area under curve [J] |
|---|---|
| 1 | 0.00000 |
