# Supplementary material for: An ingestible origami-inspired metamaterial for prolonged oral delivery of therapeutics
Source: Nat Commun. 2026 Jul 27;17:9090. doi: 10.1038/s41467-026-76028-y (PMC13518907; doi:10.1038/s41467-026-76028-y)
Supplement: Supplementary file 5 [file 41467_2026_76028_MOESM5_ESM.zip › Code-S1/fig3/L100-55_02_10_2017/DogBone_SGF_2_3.is_tens.pdf]

Quasi-static tensile test to failure.

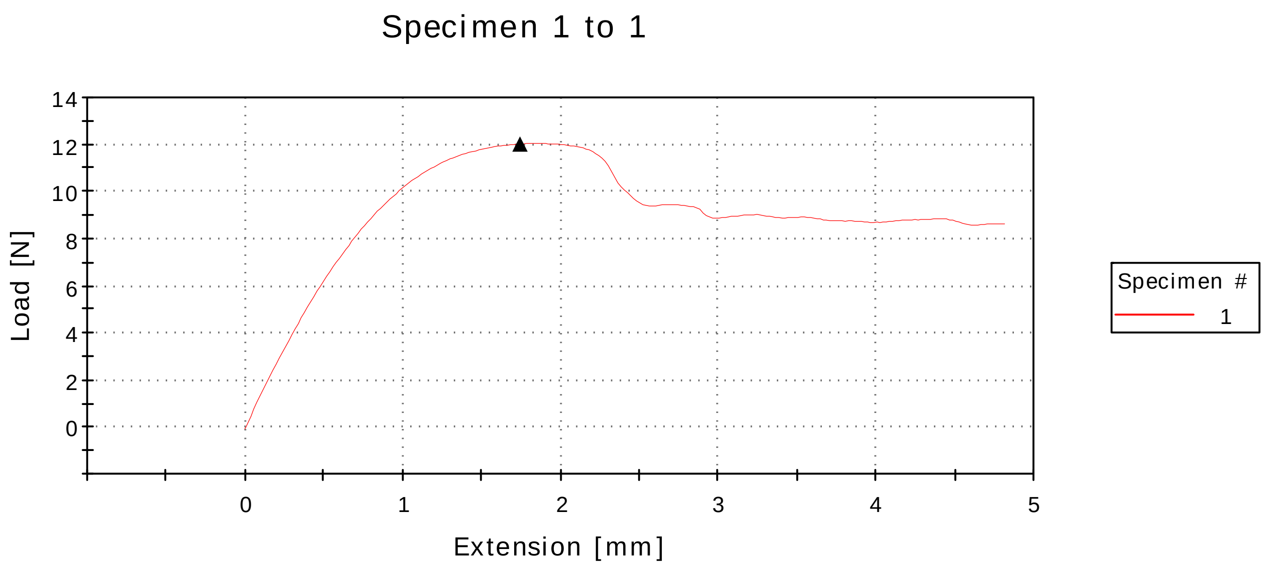

	Rate 1 [mm/min]	Time at Tensile strength [s]	Extension at Tensile strength [mm]	Load at Tensile strength [N]
1	12.00000	8.70000	1.74045	12.03421

	Tensile strain (Extension) at Tensile strength [mm/mm]	Tensile stress at Tensile strength [MPa]	Poisson's ratio (Chord)	Modulus (Automatic) [MPa]
1	0.04463	1.20342	-----	50.70774

	Area under curve [J]
1	0.00000
