# Supplementary material for: An ingestible origami-inspired metamaterial for prolonged oral delivery of therapeutics
Source: Nat Commun. 2026 Jul 27;17:9090. doi: 10.1038/s41467-026-76028-y (PMC13518907; doi:10.1038/s41467-026-76028-y)
Supplement: Supplementary file 5 [file 41467_2026_76028_MOESM5_ESM.zip › Code-S1/fig3/Test2_12_21_2016/DogBone_PCL37_Eu20__4.1.is_tens.pdf]

Quasi-static tensile test to failure.

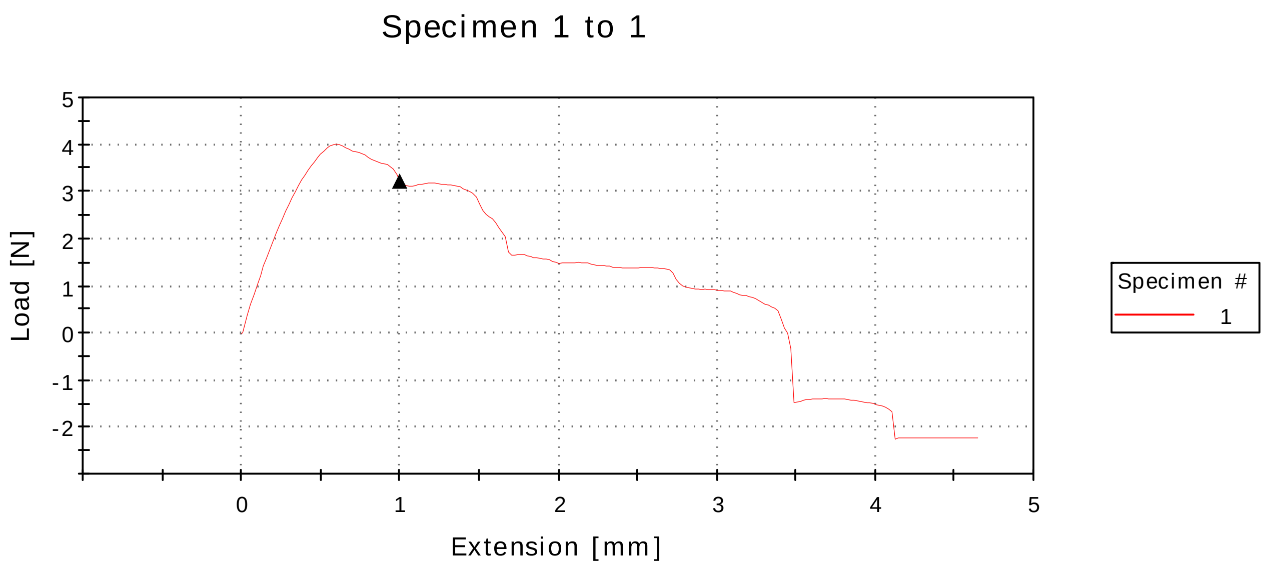

	Rate 1 [mm/min]	Time at Tensile strength [s]	Extension at Tensile strength [mm]	Load at Tensile strength [N]
1	12.00000	5.00000	1.00043	3.23604

	Tensile strain (Extension) at Tensile strength [mm/mm]	Tensile stress at Tensile strength [MPa]	Poisson's ratio (Chord)	Modulus (Automatic) [MPa]
1	0.02565	0.32360	-----	39.64695

	Area under curve [J]
1	0.00000
