# Supplementary material for: An ingestible origami-inspired metamaterial for prolonged oral delivery of therapeutics
Source: Nat Commun. 2026 Jul 27;17:9090. doi: 10.1038/s41467-026-76028-y (PMC13518907; doi:10.1038/s41467-026-76028-y)
Supplement: Supplementary file 5 [file 41467_2026_76028_MOESM5_ESM.zip › Code-S1/fig3/Test2_12_21_2016/DogBone_PCL37_Eu30__2.1.is_tens.pdf]

Quasi-static tensile test to failure.

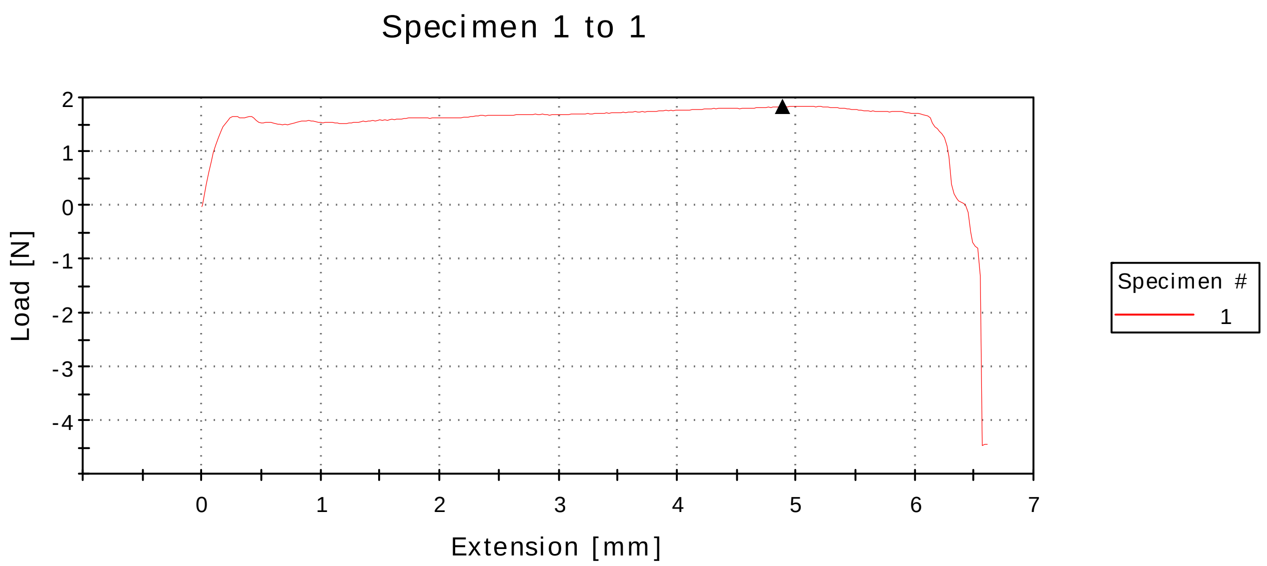

	Rate 1<br>[mm/min]	Time at Tensile strength<br>[s]	Extension at Tensile strength<br>[mm]	Load at Tensile strength<br>[N]
1	12.00000	24.40000	4.87995	1.82916

	Tensile strain (Extension) at Tensile strength<br>[mm/mm]	Tensile stress at Tensile strength<br>[MPa]	Poisson's ratio (Chord)	Modulus (Automatic)<br>[MPa]
1	0.12513	0.18292	-----	38.35864

	Area under curve<br>[J]
1	0.00000
